# Identification of monoclonal antibodies against human renal glomerular endothelial cells in lupus nephritis that induce endothelial interferon-alpha production

**DOI:** 10.1186/s13075-021-02552-5

**Published:** 2021-06-16

**Authors:** Ya-Chiao Hu, I-Jung Tsai, Hui-Yao Hsu, Bor-Luen Chiang, Yao-Hsu Yang

**Affiliations:** 1 Department of Pediatrics, National Taiwan University Hospital, College of Medicine, National Taiwan University, No. 7 Chung-Shan South Road, Taipei, Taiwan; 2 Graduate Institute of Clinical Medicine, College of Medicine, National Taiwan University, Taipei, Taiwan; 3 Department of Pediatrics, National Taiwan University Hospital, Hsin-Chu Branch, Hsinchu, Taiwan

**Keywords:** Lupus nephritis, Monoclonal antibody, Human renal glomerular endothelial cells, Interferon-α

## Abstract

**Background:**

The pathogenesis of lupus nephritis (LN) remains not fully understood. In this study, we aimed to explore the pathogenic roles of autoantibodies against human renal glomerular endothelial cells (HRGEC) in LN patients.

**Methods:**

The serum levels of anti-HRGEC antibodies in systemic lupus erythematosus (SLE) patients without LN and LN patients were determined by cell-based enzyme-linked immunosorbent assay (ELISA). Monoclonal IgG anti-HRGEC antibodies were subsequently generated from LN patients. The binding activities of these monoclonal antibodies to HRGEC, their cross-reactivity with double-stranded DNA (dsDNA), and the ability to activate HRGEC were further evaluated.

**Results:**

LN patients had higher serum levels of IgG anti-HRGEC antibodies than SLE patients without LN and healthy controls. Four monoclonal IgG anti-HRGEC antibodies (LN1–4) were obtained; LN1 and LN2 were IgG3 while LN3 and LN4 were IgG1. Among these monoclonal antibodies, LN1–3 were cross-reactive with dsDNA. The functional assays showed that compared with IgG1/IgG3 isotype controls, LN3 had an effect on HRGEC to enhance interleukin (IL)-6 production, LN4 could enhance IL-8 and monocyte chemoattractant protein (MCP)-1 production, and LN1–3 possessed the ability to induce interferon (IFN)-α production by HRGEC. Moreover, the removal of DNA on the HRGEC surface by DNAse 1 did not interpose the binding of LN1–3 to HRGEC and the effects of LN1–3 on IFN-α induction by HRGEC.

**Conclusions:**

Some IgG anti-HRGEC antibodies in LN patients had the ability to enhance endothelial proinflammatory cytokine (IL-6, IL-8, and MCP-1) production, and some could induce the DNA-independent production of IFN-α by HRGEC.

## Background

Systemic lupus erythematosus (SLE) is a complex chronic autoimmune disorder, which is most prevalent among females of childbearing age but can occur during childhood and also in males [1]. It is characterized by the breakdown of tolerance to self-antigens and the production of many autoantibodies [2]. Such immune dysregulation affects multiple organ systems. Of them, renal involvement is a major cause of morbidity and mortality [3]. Compared with adults, children with SLE are more likely to develop lupus nephritis (LN) (34–48% in adults and 50–75% in children) [4, 5]. Class IV and/or class III LN are the most frequent and severe lesions that may progress to end-stage renal disease even under aggressive treatment [3, 4].

Although LN is common in SLE, the pathogenic mechanisms are complicated and yet to be fully determined. Most patients with LN have high serum levels of IgG anti-double-stranded DNA (dsDNA) antibodies that often correlate with disease activity [6]. Some studies have shown that the administration of either human or murine anti-dsDNA antibodies to mice can induce glomerulonephritis [7–9]. However, not every SLE patient with positive anti-dsDNA antibodies has renal involvement; some patients still had high LN activity after the reduction of anti-dsDNA antibodies by rituximab therapy [10, 11]. Due to such inconsistency and uncertainty, the roles of these antibodies in the pathogenesis of LN have been extensively studied and concluded that anti-dsDNA antibodies are not always necessary for the development of LN and only part of them are pathogenic and detrimental to kidneys [6, 9, 10]. Moreover, Mannik et al. found that as many as 90% IgG eluted from kidneys of SLE patients did not bind directly to dsDNA and related nuclear components [12]. Combined, it indicates in addition to nephritogenic anti-dsDNA antibodies, there are other autoantibodies that may contribute to LN.

Anti-endothelial cell antibodies (AECA) are a heterogeneous group of antibodies that bind to different antigens on endothelial cells (EC), some of them are pathogenic and some maybe only an epiphenomenon of vascular damage [13]. AECA have been found in a variety of vascular disorders such as atherosclerosis, diabetic vasculopathy, graft rejection, vasculitis, and connective tissue diseases [14, 15]. In SLE, up to 80% of patients have been reported with positive AECA in the sera [16]. Tseng et al. found IgG AECA serum levels and anti-endothelial activities were higher in LN patients than in SLE patients without LN. Besides, IgG AECA serum levels in LN patients were well correlated with their disease activities [17]. In addition to IgG AECA, IgA AECA serum levels were also higher in LN patients and correlated with histological evidence of active lesions in LN [18]. These results revealed the association between AECA and LN but did not clarify the causal relationship.

Accordingly, we hypothesize that some AECA may have a pathogenic role in LN. We analyzed the presence of autoantibodies against EC, especially primary human renal glomerular EC (HRGEC) in SLE patients with or without LN, and subsequently generated monoclonal anti-HRGEC antibodies from some LN patients. Using monoclonal antibodies, we further evaluated the characteristics of anti-HRGEC autoantibodies and their effects on HRGEC.

## Material and methods

### Patients and healthy controls

Based on the American College of Rheumatology (ACR) and Systemic Lupus International Collaborating Clinics (SLICC) classification criteria for SLE [19, 20], 12 SLE patients with LN presented with proteinuria (> 2 g/day), hematuria, and ± cellular casts; 12 SLE patients without renal involvement; and 25 age-matched healthy controls were enrolled in the present study. The average age (in years) at the time of blood sampling from SLE patients was 17.6 (range 11–27.8). Those SLE patients with concomitant disorders such as diabetes mellitus and hypertension that may affect renal function were excluded in this study. The written informed consents were obtained from all subjects, and this study had been approved by the Research Ethics Committee of National Taiwan University Hospital.

### Enzyme-linked immunosorbent assays (ELISA) for antibodies against EC

HRGEC (ScienCell Research Laboratories, CA, USA) and human umbilical vein endothelial cells (HUVEC) (Clonetics, USA) were used for subsequent experiments between the 2nd and the 6th passage. They were seeded respectively on bovine plasma fibronectin (BPF)- and gelatin-coated 96-well microtiter plates (Nunc™, Demark) at a concentration of 1 × 10^4^ cells/well. When the cellular growth became confluent 3–4 days later, cells were fixed with 0.2% glutaraldehyde in PBS for 10 min at room temperature and blocked with 1% BSA in PBS for 60 min at 37 °C. After washing with PBS, the serum samples or monoclonal antibodies, diluted in 1% BSA/PBS as indicated concentrations, were added and incubated for 2 h at 37 °C. The sera or monoclonal antibodies were then removed, and the plates were washed; 100 μl of peroxidase-conjugated rabbit anti-human IgG, IgA, or IgM immunoglobulins was added to each well for further 2 h at 37 °C. After washing, tetramethylbenzidine (TMB) (KPL, USA) solution was added for 15 min and stop solution (1 M hydrochloric acid) for 5 min. The optical density (OD) of each well was read at a wavelength of 450 nm against a background of 650 nm in a VersaMax™ microplate reader (Molecular Device, San Jose, CA, USA).

### Generation of monoclonal antibodies against HRGEC

Monoclonal antibodies were generated as previously described [21, 22]. Briefly, peripheral blood mononuclear cells (PBMC) from four patients with LN were transformed with Epstein-Barr virus and cultured in 96-well plates. The supernatants were screened for desired IgG antibodies by HRGEC-based ELISA described above. Cells from each positive well were subcloned twice at one cell per well to yield monoclonal cell lines. Thereafter, each monoclonal EBV transformed cell line was fused with the Oubain-resistant K6H6/B5 human-mouse heterohybridoma cell line. Again, positive hybridomas were subcloned twice at 1 cell per well. To ensure the monoclonality of each monoclonal antibody, the light chain isotypes and IgG subclasses were determined by ELISA using isotype and subclass-specific reagents. To purify monoclonal antibodies, hybridomas were switched to a serum-free culture medium. Culture supernatants were passed through a HiTrap Protein G column (Pharmacia, Piscataway, NJ, USA), and the bound IgG was eluted with 0.1 M glycine HCl (pH 2.8) and dialyzed against PBS.

### Immunofluorescence staining for the binding of monoclonal antibodies to HRGEC

HRGEC were seeded on BPF-coated 24-well plates (Thermo Fisher Scientific, Waltham, MA, USA) at a concentration of 5 × 10^4^ cells/well. When the cellular growth became confluent 3–4 days later, cells were fixed with 4% paraformaldehyde (Sigma-Aldrich, St. Louis, MO, USA) in PBS for 15 min at room temperature and washed by PBS. Subsequently, the cells were incubated with a blocking buffer containing 3% BSA/PBS for 30 min at room temperature. After washing, monoclonal antibodies including patient-derived IgGs and their corresponding isotype controls (Sigma-Aldrich, St. Louis, MO, USA) (10 μg/ml) were added at 4 °C overnight. The cells were then washed and incubated with FITC-conjugated goat anti-human IgG and DAPI (Abcam, UK) for 40 min at room temperature. Finally, the cells were mounted in ProLong™ Gold Antifade Mountant (Thermo Fisher Scientific, USA) and read by an inverted fluorescence microscope (Carl Zeiss Axio Observer).

### Flow cytometry for the binding of monoclonal antibodies to HRGEC

HRGEC at a concentration of 1 × 10^5^ cells/tube were suspended with RPMI 1640 and incubated with patient-derived monoclonal antibodies or isotype controls (10 μg/ml) at 4 °C for 30 min. The cells were then washed by cold buffer and incubated with AF 488-conjugated mouse anti-human IgG (Thermo Fisher Scientific, USA/SouthernBiotech, Birmingham, USA) at 4 °C for 30 min. After washing, stained cells were re-suspended in cold staining buffer and analyzed with a FACSCalibur cell analyzer (BD Biosciences, San Jose, CA, USA).

### The reactivity of monoclonal antibodies with dsDNA and HRGEC

The binding activities of patient-derived monoclonal antibodies and IgG subclass isotype controls with dsDNA were evaluated by a commercial IgG anti-dsDNA ELISA kit containing positive and negative controls (CUSABIO TECHNOLOGY LLC, Houston, USA). According to the manufacturer’s instructions, the cutoff value was equal to the average negative control OD value + 0.1. To remove the chromatin materials entrapped on the surface of EC, in some experiments, HRGEC confluent on microtiter plates were incubated with DNAse I (40 μg/ml) and 10 mM MgCl_2_ for 1 h at 37 °C [23]. The binding affinities of each monoclonal antibody positive for dsDNA to DNAse I-treated and non-treated HRGEC were further assayed and compared by the cell-based ELISA.

### The effects of monoclonal antibodies on HRGEC activation

HRGEC were first seeded on BPF-coated 24-well plates at a concentration of 5 × 10^4^ cells/well. When the cellular growth became confluent, the supernatants were removed. Each well was then washed by PBS and incubated with serum-free Endothelial Cell Medium (ScienCell Research Laboratories, CA, USA). Patient-derived IgG monoclonal antibodies and their corresponding IgG isotype controls at different concentrations (final conc. 100 μg/ml, 50 μg/ml, 25 μg/ml, 12.5 μg/ml, 6.25 μg/ml, 0 μg/ml) were individually added to each well at 37 °C. Twenty-four hours later, the supernatants were collected for the analysis of interleukin (IL)-1, IL-6, IL-8, monocyte chemoattractant protein (MCP)-1, interferon (IFN)-γ, and IFN-α (IL-1, 6, 8; MCP-1; and IFN-γ detected by DuoSet ELISA Kits, R&D Systems, Inc., Minneapolis, USA; IFN-α detected by Matched Antibody Pair Kit, Eugene, OR, USA). Moreover, in the experiment of endothelial IFN-α production, some HRGEC were pre-treated with DNAse I. The effects of selected dsDNA-reactive monoclonal anti-HRGEC antibodies on IFN-α production by DNAse I-treated HRGEC were evaluated.

### Statistical analysis

The values in this study were presented as means ± standard deviations (SD) or means with a range. The variates including serum levels of AECA (shown as OD values) and IFN-α among the groups were compared by analysis of variance (ANOVA) followed by the Bonferroni multiple comparison test. The comparison of other parameters between LN patients and SLE patients without LN was conducted by the Student’s *t* test. The differences in cytokine production between monoclonal antibody-treated and isotype control-treated HRGEC were analyzed by the Mann-Whitney U test. A two-tailed *p* value of less than 0.05 was considered statistically significant.

## Results

### Characteristics and laboratory data of SLE patients

Twenty-four SLE patients enrolled in this study were all positive for both antinuclear antibodies (ANA) and anti-dsDNA antibodies. As can be seen in Table 1, the age distributions between SLE patients with and without LN were comparable. Those LN patients had higher anti-dsDNA antibody serum levels and lower complement (C)3, C4, and hemoglobin serum levels than SLE patients without LN. Of note, among 12 LN patients, five patients had received renal biopsies and all showed class IV diffuse proliferative glomerulonephritis. Treatments ever used for patients are shown in Table 1. More LN patients had ever received methylprednisolone pulse therapy (11/12 vs 3/12) and cyclophosphamide pulse therapy (9/12 vs 1/12) than patients without LN.

**Table 1:** Characteristics, laboratory data, and treatments of SLE patients with and without LN

	LN patients (*N* = 12)	SLE patients without LN (*N* = 12)
Female: male	10:2	11:1
Age in years	17.34 (11–27)	17.93 (12–27.8)
Laboratory data		
ANA (+)	12 (100%)	12 (100%)
Anti-dsDNA Ab (+)	12 (100%)	12 (100%)
Anti-dsDNA Ab level (IU/ml)*	790.04 ± 257.39	508.25 ± 277.07
DRVVT and/or IgG/IgM anticardiolipin Ab (+)	4 (33.3%)	3 (25%)
WBC count (× 10^3^/ml)	6292.20 ± 4097.73	5632.50 ± 2435.47
Hemoglobin (g/dl)**	9.63 ± 1.92	11.9 ± 1.36
Platelet count (× 10^3^/ml)	210.00 ± 99.10	220.17 ± 96.94
C3 (mg/ml)**	40.67 ± 18.45	76.16 ± 18.79
C4 (mg/ml)**	7.41 ± 3.78	13.52 ± 5.35
Treatments (ever used)		
Corticosteroid	12 (100%)	12 (100%)
MP pulse therapy**	11 (91.7%)	3 (25%)
CTX pulse therapy**	9 (75%)	1 ( 8.33%)
HCQ	12 (100%)	12 (100%)
AZA	5 (41.7%)	6 (50%)
CsA	7 (58.3%)	6 (50%)
MTX	3 (25%)	2 (16.7%)
MMF	5 (41.7%)	4 (33.3%)

*ANA* antinuclear antibodies, *Ab* antibodies, *DRVVT* dilute Russell viper venom time, *C* complement, *MP* methylprednisolone, *CTX* cyclophosphamide, *HCQ* hydroxychloroquine, *AZA* azathioprine, *CsA* cyclosporine A, *MTX* methotrexate, *MMF* mycophenolate mofetil

*Significant difference (*p* < 0.05) between LN patients and SLE patients without LN

**Significant difference (*p* < 0.001) between LN patients and SLE patients without LN

### Serum antibodies against HRGEC and HUVEC in SLE patients

**Fig. 1:**
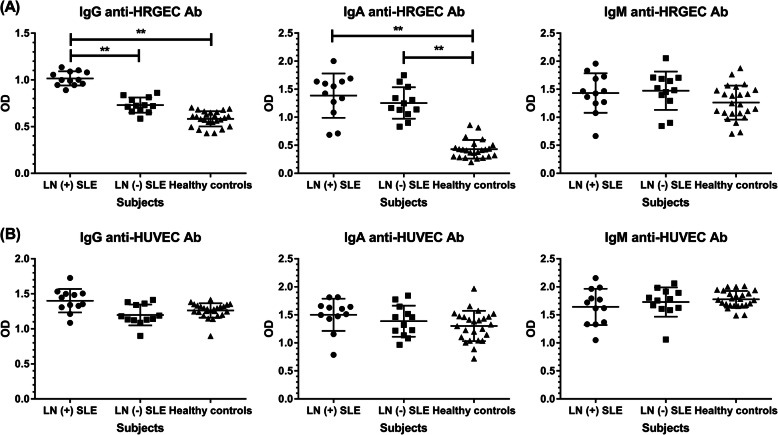
Detection of AECA in SLE. Serum samples from 12 SLE patients with LN, 12 SLE patients without LN, and 25 healthy controls were analyzed at 1: 100 for IgG, 1: 50 for IgA, and 1: 50 for IgM antibodies against **a** HRGEC and **b** HUVEC. The mean and SD are given. **p < 0.001

To test our hypothesis and elucidate the pathogenic roles of AECA in LN, we first used a cell-based ELISA for anti-HRGEC antibodies to analyze the serum samples of 12 LN patients, 12 SLE patients without LN, and 25 healthy controls. Figure 1a shows that the serum levels of IgG anti-HRGEC antibodies were significantly higher in LN patients than in SLE patients without LN (OD values 1.02 ± 0.08 vs 0.73 ± 0.08, *p* < 0.001) and healthy controls (OD values 1.02 ± 0.08 vs 0.58 ± 0.08, *p* < 0.001). Among 12 LN patients, 7 were children while 5 were adults. The serum levels of IgG anti-HRGEC antibodies were not significantly different between childhood LN and adult LN (OD values 1.03 ± 0.09 vs 1.00 ± 0.06, p = 0.536). SLE patients no matter with or without LN had higher IgA anti-HRGEC antibody serum levels than healthy controls (1.39 ± 0.40 vs 0.43 ± 0.17, *p* < 0.001; 1.25 ± 0.28 vs 0.43 ± 0.17, *p* < 0.001). However, there was no difference in IgM anti-HRGEC antibody serum levels among the 3 groups. Using a similar assay, the above samples were simultaneously evaluated for the presence of antibodies against HUVEC, the EC that are commonly used in AECA-related studies. As shown in Fig. 1b, the serum levels of IgG, IgA, and IgM anti-HUVEC antibody serum levels were not significantly different among the 3 groups. The discrepancy of presentation between anti-HRGEC and anti-HUVEC antibodies indicates each EC of different origin may have its distinct structural components and characteristics. Since this study addressed the pathogenesis of LN, HRGEC instead of HUVEC were used for subsequent experiments.

### Generation of four monoclonal IgG anti-HRGEC antibodies from LN patients

According to the above results that IgG but not IgA or IgM anti-HRGEC antibody serum levels in LN patients were significantly higher than that in SLE patients without LN, we initiated the efforts to generate monoclonal IgG anti-HRGEC antibodies from LN patients with high titers of such antibodies and finally obtained 4 monoclonal antibodies (LN1–4). To ensure the monoclonality of each monoclonal antibody, the heavy chain subclass and light chain isotype of each antibody were determined. The results showed that each antibody had only one light chain isotype and one IgG subclass. Specifically, LN1, LN2, and LN3 have λ light chains, while LN4 had κ light chains. For heavy chains, LN1 and LN2 were of the γ3 subclass, while LN3 and LN4 were of the γ1 subclass.

**Fig. 2:**
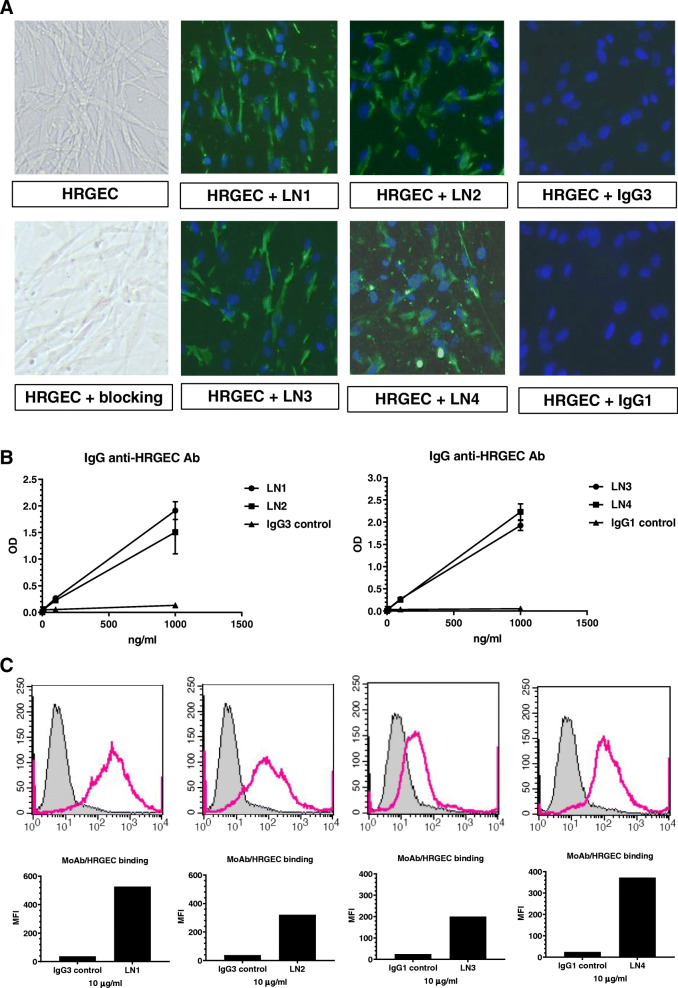
The reactivity of patient-derived monoclonal antibodies with HRGEC. **a** Immunofluorescence staining for the binding of LN1–4 to HRGEC. HRGEC were fixed with 4% paraformaldehyde, blocked by buffer containing 3% BSA/PBS, and then incubated with LN1–4 or IgG1/IgG3 isotype controls (10 μg/ml), and FITC-conjugated goat anti-human IgG. Finally, the results were detected by a fluorescence microscope (× 100). For an accurate comparison of fluorescence signals, each image was taken with the same exposure time. A representative result from 3 experiments is shown. **b** Utilizing cell-based ELISA, LN1–4 and IgG1/IgG3 isotype controls were analyzed at the indicated concentrations for their bindings to HRGEC. The mean and SD are given. **c** Flow cytometry for the binding of LN1–4 to HRGEC. HRGEC were suspended with RPMI 1640 and incubated with LN1–4 or IgG1/IgG3 isotype controls (10 μg/ml) and then incubated with AF 488-conjugated mouse anti-human IgG. Stained cells were re-suspended in cold staining buffer and analyzed with a FACSCalibur cell analyzer. One of two experiments with similar results is shown

The binding of LN1–4 to HRGEC was visualized in Fig. 2a by immunofluorescence staining. Moreover, utilizing cell-based ELISA, it was shown that LN1–4 bound well to HRGEC in a dose-dependent manner (Fig. 2b). However, the fixation of cells in both ELISA and immunofluorescence staining may induce permeabilization of EC membranes and result in the antibody response to cytoplasmic components [14]. Therefore, the binding of patient-derived monoclonal antibodies to HRGEC was further evaluated by flow cytometry, in which cells were suspended. Comparing with isotype controls, Fig. 2c showed that LN1–4 actually bound to HRGEC with higher mean fluorescence intensity (MFI).

### The cross-reactivity of LN1–4 with dsDNA

**Fig. 3:**
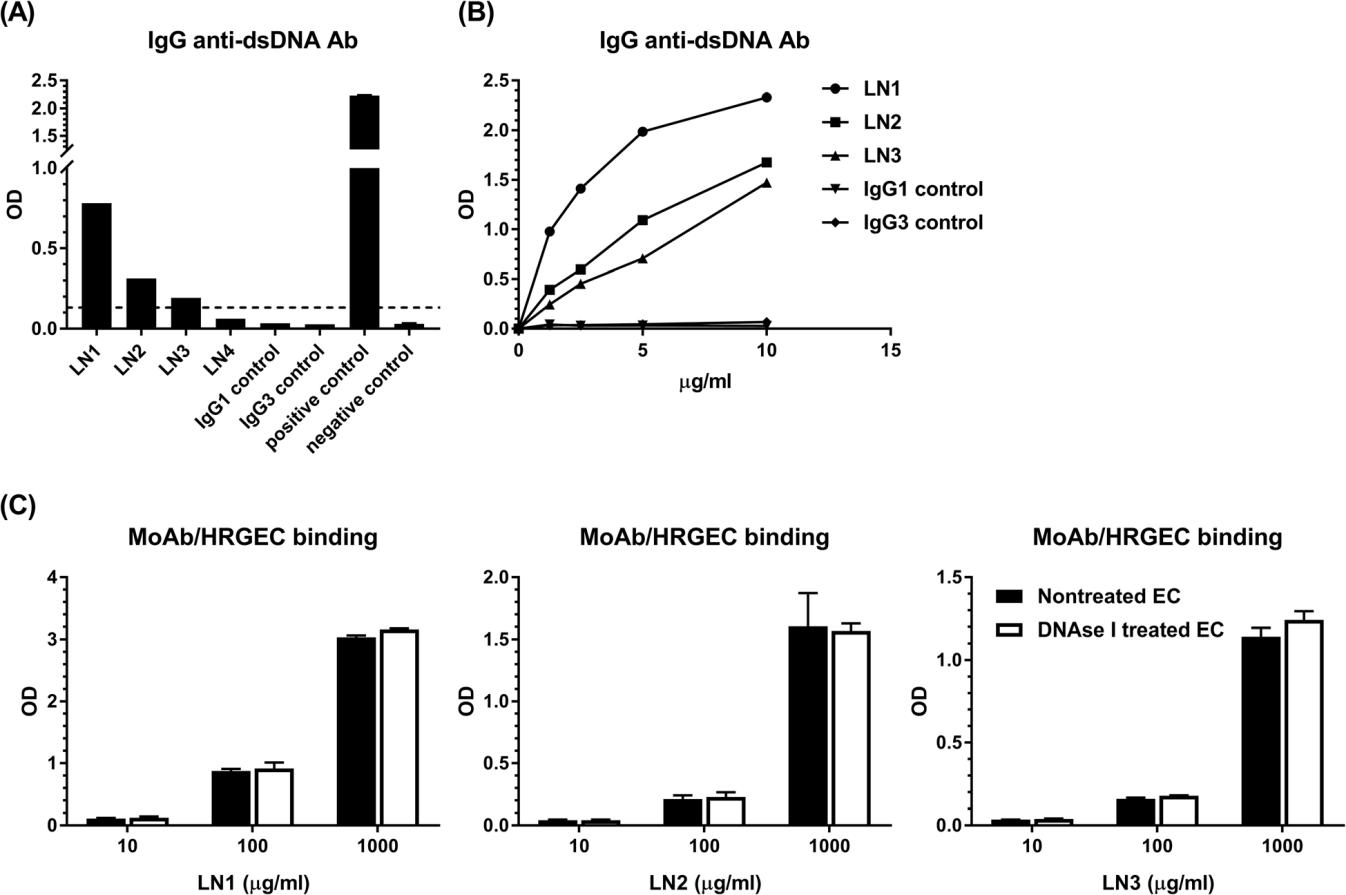
The reactivity of monoclonal antibodies with dsDNA and DNAse 1-treated HRGEC. **a** Utilizing a commercial IgG anti-dsDNA ELISA kit containing positive and negative controls, the reactivity of LN1–4 and IgG1/IgG3 isotype controls (at the indicated dilution according to the manufacturer’s instructions) with dsDNA was evaluated. The dashed line represents the cutoff, which is equal to the average negative control OD value + 0.1. One of two experiments with similar results is shown. **b** LN1–3 and IgG1/IgG3 isotype controls were analyzed at the indicated concentrations for the binding to dsDNA. One of two experiments with similar results is shown. **c** The binding activities of LN1–3 to DNAse I-treated and non-treated HRGEC were assayed by the cell-based ELISA. The mean and SD are given

Previous studies have shown that some anti-dsDNA antibodies in SLE may crossly react with EC; therefore, we further analyzed the reactivity of LN1–4 with dsDNA. Utilizing a commercial ELISA kit, we found that LN1–3 rather than LN4 and IgG1/IgG3 isotype controls bound to dsDNA (Fig. 3a). The above bindings showed a dose-dependent pattern (Fig. 3b). In a cell-based ELISA, cells such as HRGEC are seeded and cultured on microtiter plates. During the process, chromatin materials released from some apoptotic or necrotic cells may adhere to the cell surface through charge-charge interactions. Combined, a concern was raised that the generated anti-HRGEC antibodies in this study might be antibodies binding directly to dsDNA, which were first entrapped on the endothelial surface. To address this possibility, HRGEC in some experiments were treated by DNAse I to remove the dsDNA on the EC surface before the addition of monoclonal antibodies into the wells. For 3 dsDNA-reactive monoclonal antibodies, as shown in Fig. 3c, the paired binding patterns between each monoclonal antibody (LN1, LN2, or LN3) towards HRGEC and such antibody towards DNAse I-treated HRGEC were not significantly different.

### Proinflammatory cytokine production by HRGEC

**Fig. 4:**
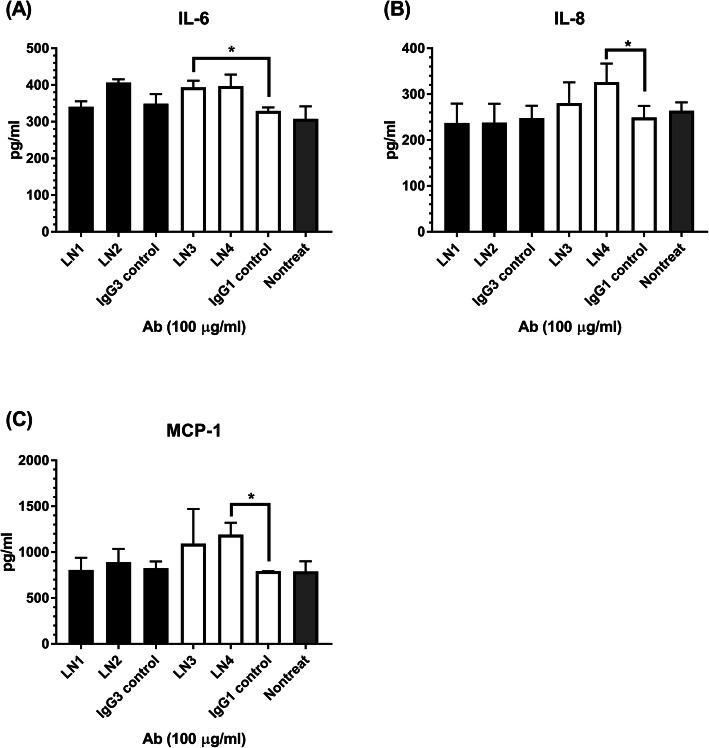
Proinflammatory cytokines produced by monoclonal antibody-treated HRGEC. The levels of **a** IL-6, **b** IL-8, and **c** MCP-1 in the supernatants of HRGEC cultured alone and co-cultured with LN1–4 or IgG1/IgG3 isotype controls at a concentration of 100 μg/ml. The mean and SD are given. *p < 0.05

LN1–4 bound directly to HRGEC, we subsequently investigate the effects of these monoclonal antibodies on HRGEC activation. Cells were cultured alone or co-cultured with LN1–4, IgG1 isotype control, and IgG3 isotype control at a final concentration of 100 μg/ml. The supernatants were then collected and analyzed the levels of proinflammatory cytokines including IL-1, IL-6, IL-8, MCP-1, and IFN-γ. No matter with or without treatment by various monoclonal antibodies, IL-1 and IFN-γ were not detected in the cell culture supernatants by the current ELISA kits. As can be seen in Fig. 4a–c, HRGEC alone can produce IL-6, IL-8, and MCP-1. The IL-6, IL-8, and MCP-1 levels between supernatants of LN1- or LN2-treated HRGEC culture and IgG3 isotype control-treated HRGEC culture were not significantly different. In contrast, compared with IgG1 isotype control, LN3 was able to enhance the production of IL-6, while LN4 enhanced IL-8 and MCP-1 production by HRGEC.

### IFN-α in SLE patients and its production by HRGEC

**Fig. 5:**
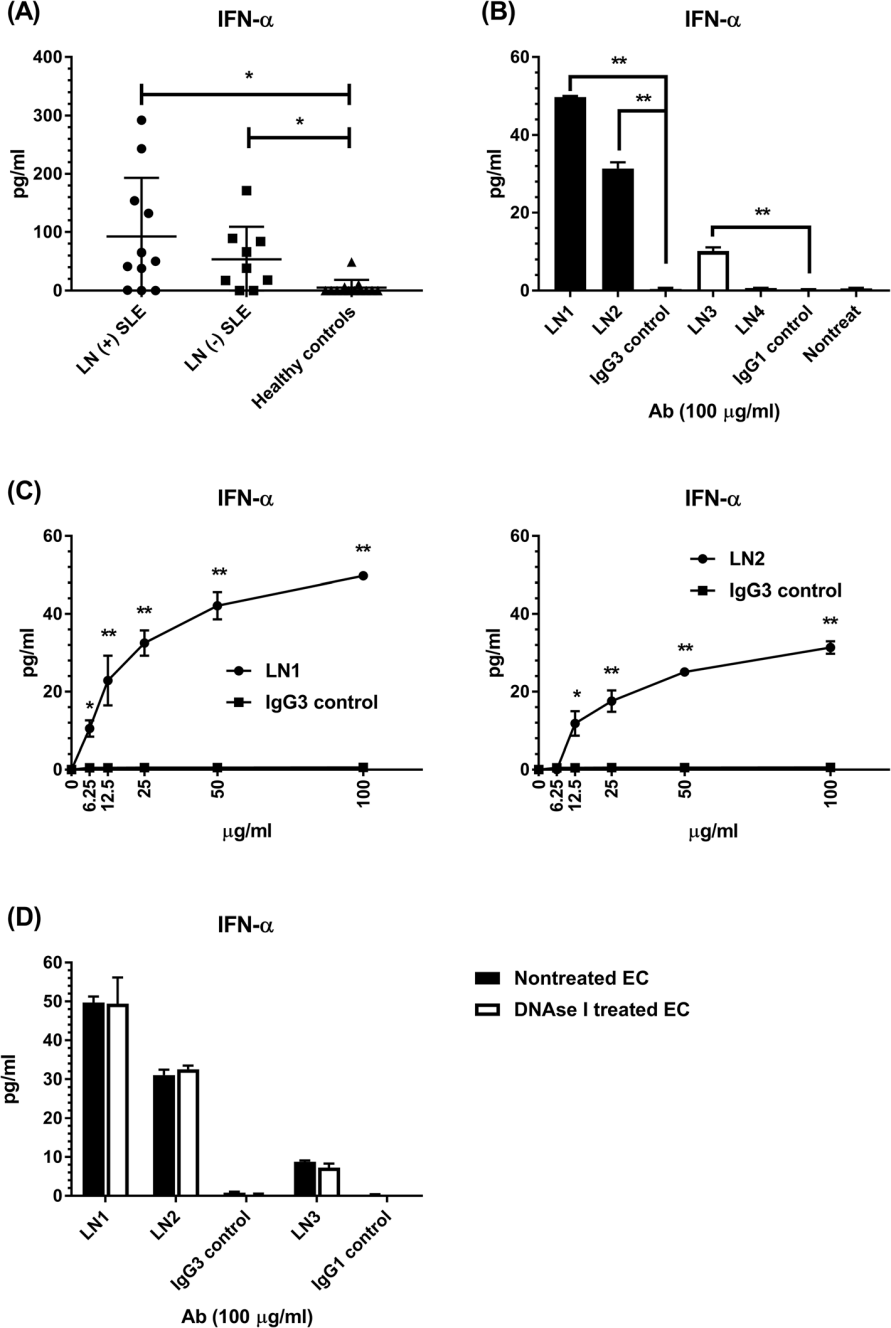
IFN-α in SLE patients and its production by monoclonal antibody-treated HRGEC. **a** Serum levels of IFN-α between SLE patients with LN, SLE patients without LN, and healthy controls. The mean and SD are given. *p < 0.05. **b** The levels of IFN-α in the supernatants of HRGEC cultured alone and co-cultured with LN1–4 or IgG1/IgG3 isotype controls at a concentration of 100 μg/ml. The mean and SD are given. **p < 0.001. **c** The levels of IFN-α in the supernatants of HRGEC treated with LN1, LN2, and IgG3 isotype control at the indicated concentrations. The mean and SD are given. *p < 0.05, **p < 0.001. **d** The levels of IFN-α between the supernatants of cultured HRGEC and DNAse I-treated HRGEC that were co-cultured with the same monoclonal antibodies (LN1, LN2, or LN3) at the same concentration of 100 μg/ml. The mean and SD are given

Since type I IFNs, particularly IFN-α, have been reported to play an important role in SLE, the serum levels of IFN-α in subjects of this study and the production of IFN-α by monoclonal antibody-treated HRGEC were evaluated. Although the serum levels of IFN-α between LN patients and SLE patients without LN were not different, both groups had higher serum levels of IFN-α than healthy controls (LN patients vs healthy controls, 92.45 ± 30.35 vs 5.05 ± 3.77 pg/ml, *p* = 0.005; SLE patients without LN vs healthy controls, 53.82 ± 18.5 vs 5.05 ± 3.77 pg/ml, *p* = 0.006) (Fig. 5a). Like IL-1 and IFN-γ, HRGEC seemed not to produce ELISA-detectable IFN-α spontaneously (Fig. 5b). Using tumor necrosis factor (TNF)-α at different concentrations to stimulate HRGEC, IFN-α was still undetectable in the cell culture supernatants (data not shown). However, it is worthy to note that LN1, LN2, and LN3 possessed the ability to induce IFN-α production by HRGEC as shown in Fig. 5b. Moreover, such endothelial IFN-α induction by monoclonal antibodies represented a dose-dependent manner (Fig. 5c).

LN1, LN2, and LN3 were crossly reactive with dsDNA. To clarify whether the induction of endothelial IFN-α production by these monoclonal antibodies is mediated through the binding towards cell surface-entrapped dsDNA, HRGEC were pre-treated with DNAse I to remove the surface DNA. Figure 5d shows that the IFN-α levels in each monoclonal antibody (LN1, LN2, or LN3)-treated HRGEC culture supernatants were not significantly different from that in the culture supernatants of DNAse I-pre-treated HRGEC that were treated by the same antibody (at the same concentration).

## Discussion

In the present study, we demonstrated the presence of IgG anti-HRGEC antibodies in LN patients. Their serum levels were higher in LN patients than in SLE patients without LN and healthy controls. Previous AECA studies in autoimmune diseases including SLE usually used HUVEC as the experimental target [14–16, 18]. Considering EC of different origin may have different characteristics [14], the binding patterns between antibodies (IgG/A/M) of subjects towards HRGEC and HUVEC were inconsistent, and this is a study focusing on LN; using HRGEC for experiments seems to be more in line with the real physiological condition. Thereafter, to further explore the roles of these antibodies in LN, we made efforts to generate human IgG monoclonal antibodies against HRGEC for subsequent functional assays but did not purify the IgG anti-HRGEC antibodies directly from the patients’ serum, which are polyclonal and functionally heterogeneous.

Anti-dsDNA antibodies are the hallmark of SLE that have been shown to contribute to systemic inflammation by the interaction with monocytes and macrophages [24]. In addition, they are also implicated in some organ involvement, particularly glomerulonephritis [6–10]. Accumulating evidence reveals that some anti-dsDNA antibodies play an important pathogenic role in LN through the binding to the surface of various resident renal cells including mesangial cells, proximal tubular epithelial cells, and glomerular EC [9]. Of 4 patient-derived IgG anti-HRGEC monoclonal antibodies in this study, 3 of them (LN1–3) were cross-reactive with dsDNA. We found that the removal of dsDNA on the cell surface by DNAse I treatment did not interfere with the binding activity of each dsDNA-reactive monoclonal antibody towards HRGEC. The results indicated that such binding of LN1–3 was independent of surface dsDNA acting as a bridge. Together with the findings of flow cytometric analysis, LN1–3 seemed to bind directly to specific antigens on the surface of HRGEC that may share compositional or conformational similarities with dsDNA.

Since AECA represent a group of EC-reactive antibodies existing in many disorders, their pathogenic mechanisms are individually different depending on the underlying disease and EC origin. We previously found that AECA of IgA isotype from patients of acute Henoch-Schönlein purpura enhanced endothelial IL-8 production, induced alternative complement activation, and also complement-dependent HUVEC lysis [25–27]. Ahmed et al. reported that there were distinct AECA subsets in patients with systemic sclerosis that induced dermal EC apoptosis and EC fibrillin-1 expression [28]. In SLE, AECA isolated from the serum have been shown to enhance the expression of adhesion molecules and the production of proinflammatory cytokines by HUVEC [29]. In this context, the effects of LN1–4 on HRGEC activation were assayed. The results showed that LN3 enhanced IL-6 while LN4 enhanced IL-8 and MCP-1 production by HRGEC. IL-6 is a pleiotropic cytokine with a wide range of biological activities that plays an important role in antibody production and inflammation [30]. IL-8 is a potent chemoattractant that induces the migration of neutrophils and lymphocytes to the sites of inflammation [31]. Besides induction of monocyte/macrophage recruitment, MCP-1 has been found to induce inflammatory activation of human tubular epithelial cells [32]. Their urine levels were correlated with the extent of proteinuria [33]. Combined, some anti-HRGEC antibodies in LN patients may enhance the local inflammation in the kidney by augmenting endothelial proinflammatory cytokine production.

More interestingly, in addition to enhancing the production of the above proinflammatory cytokines (IL-6, IL-8, and MCP-1), some of the patient-derived monoclonal antibodies (LN1–3) were found to induce IFN-α production by HRGEC. Initially, the final concentration of each monoclonal antibody for functional assays was 100 μg/ml. Assuming that a total serum IgG concentration is ~ 10 mg/ml, a concentration of 100 μg/ml represents 1% of serum IgG. Thus, the observed HRGEC activation activity of antibodies in some LN patients is not likely to be artificially exaggerated. To further determine the pathological significance of anti-HRGEC antibody-mediated induction of IFN-α production by HRGEC, we analyzed two chosen monoclonal antibodies (LN1, LN2 plus an IgG3 isotype control) at a series of 2-fold lower concentrations (from 100 to 6.25 μg/ml). The results showed that LN1 at a low concentration of 6.25 μg/ml and LN2 at a concentration of 12.5 μg/ml could significantly induce endothelial IFN-α production.

Recent advances in understanding the innate immunity in SLE have revealed the significance of type I IFNs, specifically IFN-α that not only modulates systemic autoimmunity, but also impacts LN [34–37]. Different from temporary IFN-α induction by viral nucleic acids during viral infection, the exposure to endogenous nucleic acids from dead cells in SLE results in sustained IFN-α production mainly by plasmacytoid dendritic cells (pDC) and neutrophils, and the presence of a broad IFN-inducible genes (IFIG) expression signature in these cells [34, 37]. As such, the current and previous studies demonstrated that IFN-α serum levels in SLE patients were higher than healthy controls [34, 38]. In addition, the expression of IFIG within PBMC of SLE patients has been detected and found to be associated with disease activity [35, 39]. Abundant pDC infiltrate was found in the kidneys of LN patients, and also the IFN-α transcripts in their renal biopsy specimens [35, 37, 40]. Besides intrarenal pDC, some in vivo and ex vivo murine studies have shown that resident renal cells such as mesangial cells and glomerular EC could produce a large amount of type I IFNs [41–43]. The IFN-α production and signaling no matter in pDC or other resident renal cells are majorly triggered by the interaction between Toll-like receptors and nucleic acids or immune complexes containing nucleic acids [34–37]. In this study, we found that some human anti-HRGEC antibodies (LN1–3) activated HRGEC to produce IFN-α. Of note, these antibodies were cross-reactive with dsDNA. Nevertheless, the above activation ability was not abrogated after the removal of DNA on the HRGEC surface. Although more studies are needed, the results indicated such antibodies may trigger HRGEC to secrete IFN-α through a DNA-independent pathway.

SLE is now characterized as an independent risk factor for vascular endothelial dysfunction that is associated with various comorbidities including LN [44]. Several studies addressing the effects of IFN-α on EC of different origin have shown that IFN-α inhibited the endothelial repair, reduced the transcription of endothelial nitric oxide synthase (eNOS), reduced the eNOS cofactor availability, and increased reactive oxygen production, which may collectively lead to endothelial dysfunction [44–46]. Moreover, data from murine LN models have demonstrated that IFN-α damaged the podocytes and induced chemokines that are responsible for the recruitment of inflammatory cells, particularly neutrophils and monocytes to the kidneys [34, 43]. Together, it is conceivable that the local production of IFN-α induced by some human anti-HRGEC antibodies may contribute partly to the development of LN.

There are some limitations of this study; this is an in vitro study; in addition, the signaling pathway through which anti-HRGEC antibodies induce the production of endothelial IFN-α and the epitopes that such antibodies bind to is not yet clarified. Therefore, although some LN patients had IgG anti-HRGEC antibodies, it would be problematic to ascertain the clinical significance through the association study of the presence of total IgG anti-HRGEC antibodies to LN patients. Certainly, it will be necessary to first identify the differential epitopes that are only recognized by the pathogenic anti-HRGEC antibodies (like LN1–4) and then develop a more specific assay for the detection of pathogenic anti-HRGEC antibodies in LN patients that may be helpful in the disease diagnosis and follow-up.

## Conclusions

**Fig. 6:**
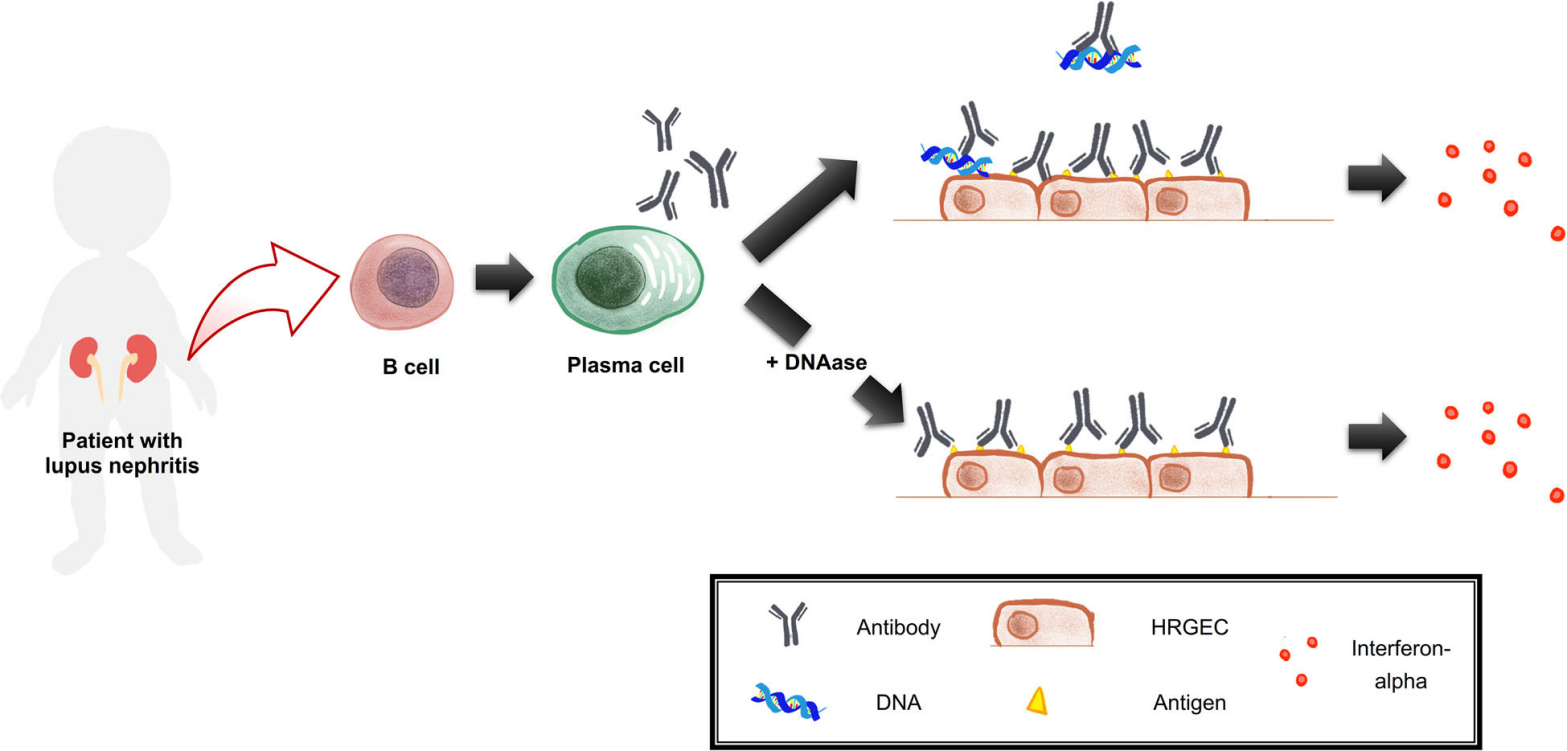
The summary of some IgG DNA-reactive anti-HRGEC antibodies in lupus nephritis inducing DNA-independent production of IFN-α by HRGEC

In summary, the current results showed some IgG antibodies in LN patients were reactive with HRGEC. Of the LN patient-derived monoclonal antibodies against HRGEC, LN3 and LN4 had the ability to enhance endothelial proinflammatory cytokine (IL-6, IL-8, and MCP-1) secretion. More importantly, LN1, 2, and 3 could induce the DNA-independent production of IFN-α by HRGEC (Fig. 6). These findings provide additional insight for a better understanding of the pathogenesis of LN.

## Data Availability

The datasets used and/or analyzed during the current study are available from the corresponding author on reasonable request.

## Abbreviations

LN: Lupus nephritis
HRGEC: Human renal glomerular endothelial cells
SLE: Systemic lupus erythematosus
ELISA: Cell-based enzyme-linked immunosorbent assay
dsDNA: Double-stranded DNA
IL: Interleukin
MCP: Monocyte chemoattractant protein
IFN: Interferon
AECA: Anti-endothelial cell antibodies
EC: Endothelial cells
HUVEC: Human umbilical vein endothelial cells
PBMC: Peripheral blood mononuclear cells
OD: Optical density
SD: Standard deviations
ANA: Antinuclear antibodies
C: Complement
MFI: Mean fluorescence intensity
TNF: Tumor necrosis factor
pDC: Plasmacytoid dendritic cells
IFIG: IFN-inducible genes
